# Integration of COVID-19 testing into existing ILI surveillance system in Ghana

**DOI:** 10.1371/journal.pgph.0005788

**Published:** 2026-01-21

**Authors:** Ernest Kenu, Delia Akosua Bandoh, Ivy Asantewaa Asante, Michael Adjabeng, Abdul Gafaru Mohammed, Donne Kofi Ameme, Joseph Asamoah Frimpong, Dennis Odai Laryea, Franklin Asiedu-Bekoe

**Affiliations:** 1 Ghana Field Epidemiology and Laboratory Training Programme, University of Ghana School of Public Health, Accra, Ghana; 2 Department of Virology, Noguchi Memorial Institute for Medical Research, Accra, Ghana; 3 Independent Researcher, Accra, Ghana; 4 Public Health Division, Ghana Health Service, Accra, Ghana; 5 Disease Surveillance Department, Ghana Health Service, Accra, Ghana; University of Michigan, UNITED STATES OF AMERICA

## Abstract

The similarities between the presentations of Influenza-Like Illness (ILI) and COVID-19, generated calls for the integration of COVID-19 surveillance into the existing ILI surveillance system. The aim was to optimize outcomes through efficient use of resources. Ghana responded to the World Health Organization call to integrate the two systems. We share the processes undertaken to integrate SARS-CoV-2 into the ILI surveillance system in Ghana. Stakeholders from Disease Surveillance Department of Ghana Health Service, National Influenza Center and the University of Ghana School of Public Health were engaged to understand the existing ILI surveillance system and determine leverage points for integration. Forms, processes and reports for both COVID-19 and ILI were reviewed and modified. Additional symptoms were added to the ILI case investigation form and risk of exposure section modified to COVID-19. Also, all ILI specimens received in the laboratory were tested for SARS-CoV-2 as well. The integrated system was piloted in 24 sites from April – June 2021 and subsequently rolled out to all sentinel sites across the nation. Integration of the ILI/SARS-CoV-2 surveillance system was rolled out to all 29 sentinel sites in June 2021. Samples collected from the sentinel sites were transported and received by the lab within 48 hours and upon receipt were tested within 24 hours. From June 16 to September 10, a total of 1520 suspected influenza samples were received and tested for SARS-CoV-2 of which 297 tested positive for SARS-CoV-2. The flexibility of the ILI surveillance system in Ghana aided the integration of the COVID-19 surveillance into the existing system. Prudent supervisory measures should be instituted by the Ghana Health Service and the National Influenza Center to ensure sentinel sites submit samples on weekly basis for testing.

## Introduction

Coronavirus disease 2019 (COVID-19), which is caused by infection with the severe acute respiratory syndrome coronavirus 2 (SARS-CoV-2), presenting with symptoms of fever, sore throat, shortness of breath which are common flu-like symptoms [1]. Globally, over 775 million cases of COVID-19 have been reported with 7.1 million deaths as at June 30 2024 [2]. Ghana confirmed its first two cases on March 12, 2020, and has since reported more than 172,052 cases and 1462 COVID-19 deaths as at June 30, 2024 [2]. COVID-19 testing is significant for identifying and isolating positive cases and containing the spread of the virus [3,4]. Countries that have implemented widespread laboratory testing for COVID-19 are able to detect and contain the virus compared to countries not conducting COVID-19 testing [5,6]. In Ghana, COVID-19 surveillance activities were initiated in December 2019 prior to case detection in the country [7]. COVID-19 readiness assessment was conducted, and a response strategy developed, led by the National Disease Surveillance Department of Ghana Health Service. The Noguchi Memorial Institute for Medical Research was the first and sole site for testing COVID-19 in the early days of the pandemic. The testing was subsequently expanded to all 16 regions of the country [8].

The sustainability of COVID-19 testing has become imminent, considering its impact on the control of the pandemic. To sustain COVID-19 testing in Ghana, there is a need to leverage on existing health/ surveillance systems particularly influenza-like illness (ILI) surveillance system due to the similarity in presentations of both conditions. This can be achieved by amplifying and redirecting the existing system toward the new pathogen, enhancing response activities through contact tracing, and building laboratory testing capacity [9,10].

The operation of the ILI surveillance system in Ghana is sentinel based. To better understand the epidemiology of ILI, monitor circulating influenza strains, and obtain information on the national activity of influenza, the ILI surveillance system was introduced in 2007 in Ghana by the Ghana Health Service (GHS) and the Noguchi Memorial Institute for Medical Research (NMIMR) in collaboration with World Health Organization (WHO), the US Centers for Diseases Control, and the US Naval Medical Research Unit No.3, (now US NAMRU EURAFCENT) [11,12]. Sentinel surveillance provides an efficient means for the timely collection of quality information to provide complete data on some of the epidemiological characteristics of severe acute respiratory infection (SARI) and influenza-like illness [11].

To help in isolating and identifying as fast as possible any new strains of the influenza virus and to use the information for control and mitigation of the impacts of influenza in humans, a robust ILI surveillance system was needed. The fact that ILI and COVID-19 share common signs and symptoms as well as similar sample collection, transportation and testing procedures has generated calls for the integration of COVID-19 surveillance into the ILI surveillance system with the view of optimizing outcomes through efficient use of resources. This paper describes the integration of COVID-19 testing into the existing ILI/SARI surveillance system in Ghana.

## Methods

### Ethics statement

Integration of the COVID-19 into the existing ILI/SARI surveillance system did not require approval since if forms part of the National surveillance strengthening activities and was done under the national public health emergency protocols established in response to the COVID-19 pandemic However, assessment of the existing surveillance was conducted as part of COVID-19 response activities in Ghana and received ethical approval from Ghana Health Service Ethics Committee (GHS-ERC 006/05/20). All stakeholders consented to be part of the activity. All data collected at any point in the assessment was kept confidential and participants assured of confidentiality.

In the pilot study, testing of ILI cases was done under the national public health emergency protocols established in response to the COVID-19 pandemic. Given the need for this integration and processes not requiring individual level participation, individual consent for SARS-CoV-2 was not a requirement; however, appropriate permissions were obtained from the national level and all verbal consent was obtained when required. Verbal consent was sought by explaining the purpose of the assessment to the health worker to be interviewed in the present of their work colleague in accordance with the IRB regulations. All their questions and concerns were fully addressed before they were engaged in the assessment. The sentinel sites for routine national influenza virus surveillance in Ghana were set up in collaboration with the Ghana Health Service as well as the Ghana Armed Forces as part of the Integrated Disease Surveillance and Response (IDSR) system of the Ghana Health Service, which is the health delivery division within the Ministry of Health. All procedures were performed according to relevant guidelines and regulations. Data was anonymized before use. Data was kept on a password-protected computer and backed up daily. Authors did not have access to the laboratory data at the NIC. Only already analysed laboratory report outputs for April, May and June without identifiers were made available to authors on a monthly basis (on the 8^th^ of the new month).

### Steps for integrating the ILI/SARS-CoV-2 surveillance system

The team outlined the measures to be followed in implementing the integrated ILI/SARS-CoV-2 surveillance system. The steps agreed included; stakeholder engagement, assessing the existing ILI system for gaps and leverage points for integration, adapting the COVID-19 testing procedures into the ILI system, piloting and subsequent implementation of the COVID-19 testing into the ILI system.

### Stakeholder engagement

The Surveillance Strategy of the Enhanced Strategies to Promote and Improve Health Securities in Ghana held a meeting with the National Influenza Center (NIC), the Surveillance Department of Ghana Health Service and the University of Ghana in February 2021 to evaluate the existing ILI surveillance system in Ghana and discuss the integration process. Members were put into groups to study and review the existing system based on the evaluation and make inputs/proposals into how the system could be revised to incorporate the reporting of COVID-19. Each group, after careful deliberations reported on what had been identified and presented suggestions on how the ILI/SARI surveillance system could be revised to integrate COVID-19 testing.

#### Assessment of the existing ILI system for gaps and leverage points for integration.

Before the integration process, there was a need to understand the performance of the ILI surveillance system to identify gaps in its operation and leverage points for the integration. Though surveillance system evaluations are part of the regular activities of the health system, a surveillance system evaluation was done in consultation with the nation, regional and district health directorates. This evaluation was done as a cross-sectional study involving stakeholders of the surveillance system across the country from 1^st^ to 12^th^ March 2021. We conducted the evaluation of the ILI surveillance system in Ghana, collecting data from eight sentinel sites across the country, 4 in the southern zone, 2 in the middle zone, and 2 in the northern zone. The essence of the evaluation was explained to stakeholders and verbal consent was obtained from them before the evaluation was carried out. For this evaluation, the ILI system operators from selected sentinel sites across the country and the National Influenza Center (NIC) were interviewed on surveillance procedures, abstracted and reviewed available ILI surveillance record for the period 2016–2020 and observed surveillance procedures at the sites. Sentinel sites were also visited to observe the system’s operations at the various levels. At the sites, data on the operation of the surveillance system was collected and we assessed the attributes of the surveillance system.

The system was assessed using the CDC updated guidelines for evaluating public health surveillance systems. The guideline entailed assessing the usefulness of the system and its key attributes; flexibility, data quality, acceptability, simplicity, sensitivity, representativeness, stability, timeliness and positive predictive value of the surveillance system [13]. In assessing the usefulness of the surveillance system and the extent to which the system meets its set objectives, the ILI/SARI surveillance system’s objectives were reviewed, and surveillance department heads were interviewed on various outbreaks that had been detected, the availability of an ILI/SARI threshold and emergence of new influenza strains per positive results received. Also, key stakeholders were interviewed regarding public health actions initiated as a result of ILI/SARI surveillance activities. Information gathered during the surveillance system evaluation was analysed and presented as a comprehensive report. Attributes were analysed by scoring.

The nine attributes were assessed using indicators from the CDC updated guidelines for evaluating surveillance systems. An indicator is scored 1 if the key finding from the evaluation supported the indicator assessed in relation to the attribute and scored 0 if the key finding was not in support of the indicator assessed in relation to the attribute. The assessment scores for the indicators were then summed and divided by the total number of indicators used in evaluating each attribute.

Overall, we scored the attributes as follows: the attribute was categorized as having a “good” performance if its score was two-thirds or above, and given a value of “3.” Above one-third but below two-thirds, we categorized it as “adequate” with a score of “2.” At or below one-third, we categorized it as “poor” performance with a score of “1.” This analysis tool was adapted 198 from a similar the work done by Nuvey and colleagues [14].

### Operations of the influenza-like (ILI) and severe acute respiratory illnesses (SARI) surveillance system

**Fig 1:**
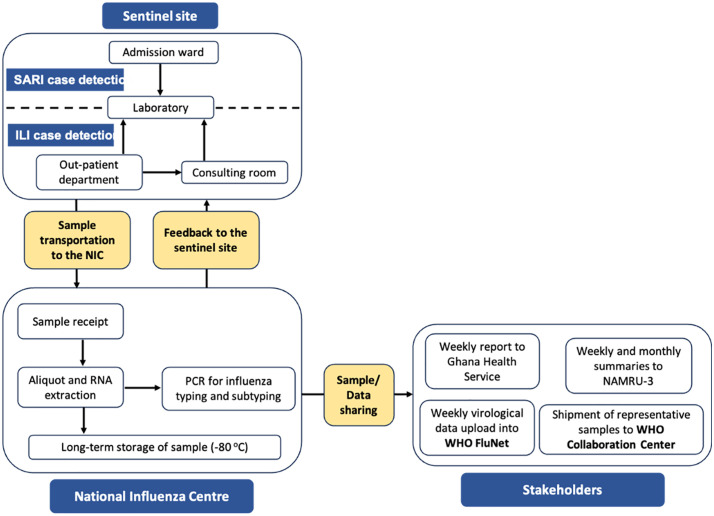
Operations of the influenza-like (ILI) and severe acute respiratory illnesses (SARI) surveillance system in Ghana.

The sentinel sites for routine national ILI/SARI surveillance in Ghana were set up in collaboration with the Ghana Health Service (GHS), the Noguchi Memorial Institute for Medical Research (NMIMR) and the Ghana Armed Forces (GAF) as part of the Integrated Disease Surveillance and Response (IDSR) system of the GHS which is the health delivery division within the Ministry of Health. Sentinel sites are comprised of regional hospitals, municipal hospitals, district hospitals, polyclinics and health centers as well as military medical reception stations (MRS) located across Ghana. At each sentinel site, there is a team composed of at least one disease control officer, a physician, nurse and laboratory personnel. Team members are stationed at the outpatient department, consulting rooms, wards and laboratory. Using the WHO case definitions for ILI/SARI (ILI: Influenza-like illness case definition: Acute respiratory infection with measured fever of 38°C or more, AND cough; with symptom onset within the past 10 days, without any age restriction, SARI: Acute respiratory infection with history of fever or measured fever of >38°C AND cough AND onset within the last 10 days and requires hospitalisation, without any age restriction), nurses at the OPD and wards identify cases, and upon confirmation, referred to the laboratory for sample collection. Patient demographic information and clinical information were collected using a case-based form. Combined oro and nasopharyngeal swab specimens are subsequently collected and transported to the NMIMR, which houses the WHO recognized National Influenza Centre (NIC) for Ghana for laboratory investigations. Received samples are analysed for the presence of influenza viruses and SARS-CoV-2 using real time reverse transcription polymerase chain reaction (rRT-PCR) with specific influenza/SARS-CoV-2 (Flu/SC2) multiplex primers from the US CDC. Samples which are positive for influenza specimens are further characterized for influenza A subtypes (AH3, A H1N1 pdm09) and B lineages (Victoria and Yamagata). Results are shared with sentinel sites and various stakeholders, including WHO, GHS and NAMRU-3. Per WHO conditions for maintaining NIC status, data is shared with WHO Global Influenza Surveillance and Response (GISRS) platforms, FluNet. Representative samples are also shared with WHO collaborating centres for influenza in Atlanta USA and the Crick Institute, UK (Fig 1). Governance of the sentinel sites is by Disease Surveillance Department, GHS and the Public Health Division of the GAF. Concurrently, case-based form has been transitioned onto an online data collection system, Surveillance Outbreak Response Management and Analysis System (SORMAS). Due to this, samples are received accompanied by a line list.

Fig 1 illustrates operations of the influenza-like (ILI) and severe acute respiratory illnesses (SARI) surveillance system in Ghana. Suspected cases are identified by members of the surveillance team at the sentinel sites. Samples are subsequently collected and sent to the NIC for analyses.

#### Adaptation of COVID-19 testing procedures into the ILI surveillance.

The similarity between COVID-19 and ILI is a precursor for the integration of the two systems. However, stakeholders upon deliberations agreed some indicators must be clearly outlined. These indicators included: the case definition, the sampling process, specimen collection and testing, the type of epidemiological data to collect and the frequency of reporting the disease.

#### Piloting and implementation of the integrated ILI/SARS-CoV-2 surveillance system.

After carefully incorporating suggested steps for integration and addressing all challenges with the existing ILI surveillance system, a pilot integrated ILI/SARS-CoV-2 surveillance system was rolled out to ensure all samples from selected ILI sentinel sites were tested for both ILI and COVID-19. The system was piloted from April to June in 24 sentinel sites across the country. After piloting the integrated ILI/SARS-COV-2 surveillance system, it was subsequently rolled out in all 29 sentinel sites.

#### Pilot study.

The pilot study aimed to assess the feasibility of integrating SARS-CoV-2 testing into the existing influenza surveillance system. To achieve this aim, sites were selected based on their: (1) historical performance in the influenza surveillance system, which includes averaging at least one sample per week with sample submission frequency not zero for more than three consecutive weeks; (2) geographic and demographic diversity, examining how integration might work under different epidemiological and healthcare delivery conditions; and (3) data reporting performance, which takes into account experience in timely and accurate reporting in the influenza surveillance system. Of the 29 sentinel sites, 24 sites were eligible and had a proven performance record. All samples that were collected during this time, were tested for both influenza and SARS-CoV-2 across all selected sites. Findings from the pilot study provided valuable insights which facilitated the expansion to the rest of the sentinel sites. Table 1 summarizes timelines for the integration.

**Table 1:** Summaries of the timeline for the integration of SARS-CoV-2 testing into the influenza surveillance system (February – July, 2021).

Activities perform for the pilot study		2021					
		Feb	Mar	Apr	May	Jun	Jul
**Preparation and training**	Prepare necessary documentation for the study						
	Engage stakeholders and conduct briefing and orientation						
	Training of health staff and access laboratory capacity						
	Testing the data reporting system (SORSMAS) for dual pathogen testing						
**Implementation**	Data and specimen collection commences						
	Accessing turnaround time from case detection to laboratory reporting (5 days)						
	Providing feedback to facility						
	Supervisory visit and midpoint review						
**Monitoring and evaluation**	Initiate preliminary data analysis						
	Conduct qualitative and quantitative checks						
	Perform final analysis						
	Prepare final report, with recommendations to scale-up						
	Debriefing meeting						

#### Supervisory visits during implementation.

Supervisory visits to the 29 sentinel sites were carried out at the time of implementation. A questionnaire was developed, focusing on the four thematic areas. These thematic areas assessed included screening and case identification, specimen collection and transport, data audit, and laboratory compliance and turnaround time. The team responsible for the exercise consisted of at least two members. The regional and district health directorate were briefed on the exercise, as well as the management teams at the health facilities, including core members of the influenza surveillance team. After going through the questionnaire, interviewing the core influenza team members (comprising a physician, nurse, disease control officer and laboratory scientist), an action plan was developed based on the gaps identified with immediate effort.

## Results

### Assessment of the existing ILI system for gaps and leverage points for integration

**Simplicity:** Case detection at the sentinel sites using the case definition was somewhat easy. However, collection and transportation of nasopharyngeal and oropharyngeal samples required trained personnel and specialized equipment. No testing of the samples was done at the sentinel sites. This attribute should a leveraging point for integration since both case definitions had similarities, sample collection was the same and testing was done offsite by the NIC.

**Flexibility:** The NIC used the ILI surveillance to detect COVID-19 by testing ILI samples received from the sentinel sites for SARS-CoV-2. This attribute showed how both systems could be integrated and still continue to run smoothly since the sample samples could be tested for both conditions.

**Data quality:** Of the 8 sentinel sites visited, 4 had sentinel registers, of which only 2 of these registers were completed filled. Out of 240 sampled case investigations forms from 4 sentinel sites, about 25% (60/240) were completed. This attribute reviewed a gap in the quality of data being generated by the existing surveillance system which had to be addressed to facilitate a smooth integration and proper functioning of the system.

**Acceptability:** Per recommendations by the NIC, each sentinel site is expected to detect up to five suspected cases of ILI every week (maximum of 260 suspected cases per sentinel site per year). Of all the sites visited who had data on cases suspected and confirmed, the Sunyani Municipal hospital met the case detection quota for all years under assessment except in the year 2020. Interview with key informants at the sites visited indicated that all the sentinel sites stopped reporting cases in the wake of COVID-19. This attribute shows a point for leveraging of integration as COVID-19 reporting and ILI reporting are through the same route.

**Sensitivity:** The data generated by the surveillance system indicated a general rise in case detection from 2016 to 2018 and a sharp decline in the number of cases confirmed from 2018 to 2020 in the various sentinel sites visited. This attribute reveals another point of leveraging since the system is sensitive enough to detect case.

**Positive Predictive Value:** = 9.9%

**Representativeness:** From the data at the sites, cases were of all ages and from both sexes. Also, all 8 sites reported on cases. From the NIC, many of the sites had not been reporting cases especially during the ongoing COVID-19 pandemic. This attributed reveals a gap in the system which had been created by the pandemic. Therefore, integration could lead to sites reporting on both conditions.

**Timeliness:** Sentinel personnel indicated that they send samples and case investigation forms on average twice each week to the NIC. A key informant interview at the NIC revealed that it takes an average of 3 days to process and test samples, and results are sent by email to facilities once ready. Analysis of ILI surveillance data showed that it took an average of 10 days from onset of symptoms for suspected cases to report for treatment in health facilities. This attribute revealed a gap in the system which needed to be addressed before the integration.

**Stability:** The NIC, the hub where data generated from ILI surveillance activities is stored, has a robust data management policy. The situation, however, was different at the sentinel sites. Only the Achimota site had a working computer and required accessories, situated in the Disease Control Unit, to conduct ILI surveillance. Funding for ILI surveillance is mainly by NAMRU-3, US CDC and WHO.

In summary, the surveillance system evaluation identified that The ILI surveillance system is very sensitive and flexible as it is able to detect cases and adjust to accommodate other events such as SARI and COVID-19. Major weaknesses in the ILI surveillance system were poor data quality at the sentinel sites visited. Most of the sentinel sites were not keeping records of samples collected, cases suspected and confirmed. Data on these were retrieved from case investigation forms where they were available and email trails. Steps were taken to address the identified gaps and points for leveraging considered during the integration process.

A workshop with stakeholders and representatives from all the sites was conducted to modify the existing reporting form to incorporated SARS-COV-2 testing. This was followed by trainings were on data quality, filling of case-based forms, reporting into SORMAS and need for proper documentation at each site.

Additionally, guidelines for integration steps were developed by stakeholders and implemented at the NIC and at all sites.

### Utility of the ILI surveillance system in meeting its set objectives

The data generated by the ILI surveillance system in Ghana is fed into the WHO FluNet database, which allows for the publication of weekly bulletins by the WHO on the circulating influenza strains and trends.

Based on the system evaluation, virtual and in-person stakeholder engagements were held to come up with a workplan and guiding document for the integration, timelines for the processes carried out during the pilot and national roll out are detailed in Table 1.

### Steps for integrating the ILI/SARS-CoV-2 surveillance system

After key stakeholder engagements, various steps were outlined for the efficient and adequate integration of COVID-19 testing into the ILI surveillance system. The following were the agreed steps for the integration.

### Expansion of the existing testing sites for ILI

The National Influenza Center at the Noguchi Memorial Institute is the WHO recognized laboratory which tests ILI samples. All samples taken across the 16 regions of the country are transported to the NIC for testing. To integrate COVID-19 testing into the existing ILI system, stakeholders agreed there is the need to introduce more testing sites so as not to put pressure on the NIC leading to a backlog of samples. Stakeholders agreed that regional public health reference labs and veterinary laboratories should be equipped to handle ILI and COVID-19 samples from their regions. This will boost the testing system and also reduce the waiting time for test results.

**a. Adaptation of COVID-19 testing procedures into the ILI surveillance**

The similarity between COVID-19 and ILI is a precursor for the integration of the two systems. However, stakeholders upon deliberations agreed some indicators must be clearly outlined. These indicators included; the case definition, the sampling process, specimen collection and testing, the type of epidemiological data to collect and the frequency of reporting the disease.

**i. Case definition:** The case definition for ILI and COVID-19 are similar and hence a person meeting the suspected or clinical case definition for one of them will be qualified for the other.
**ii. Sampling Procedures:** Within the existing surveillance system, the patients selected for testing for COVID-19 should preferably be representative of the population and include all ages and both sexes. If possible, continue to collect samples from both ILI and SARI sentinel sites to represent both mild and severe illnesses. It is recognized that based on the local situation, resources, and epidemiology, countries may wish to prioritize sampling among inpatients (SARI or pneumonia cases) to understand COVID-19 circulation in patients with more severe disease.
**iii. Specimen Collection:** The collection of nasopharyngeal or oropharyngeal specimens in ambulatory patients and/or from the lower respiratory tract (endotracheal aspirate or bronchoalveolar lavage) in patients with more severe respiratory disease is recommended for the detection of influenza virus. The team proposed the specimen collected and process used should be maintained. Storage of clinical specimens at the site of collection and transport to the testing laboratory will remain unaltered.
**iv. Laboratory Testing:** The WHO recommends using real-time reverse transcriptase-polymerase chain reaction (rRT-PCR) for laboratory confirmation of SARS-CoV-2 and influenza virus. All specimens should be tested for influenza virus and SARS-CoV-2 [10]. To detect a positivity rate of at least 2%, the WHO recommends that a sampling strategy be developed that will result in a minimum of 50 specimens per week for COVID-19 testing.
**v. Epidemiological Data Collection:** The case investigation form for ILI will need to change to accommodate epidemiological data for COVID-19 detection. The team agreed on the following modifications. The section on signs and symptoms should be reviewed to include symptoms such as headache, runny nose and a space provided for listing of other symptoms, previously not captured. The risk of exposure section should be modified to include; travel history, COVID-19 vaccination status, list of places visited during the past 7–14 days and any contact with confirmed or suspected COVID-19 patients. The team further agreed that, the laboratory investigation and results section of the form should have a column for COVID-19 investigation and results and can be sectioned as 6a for ILI and 6b for COVID-19. A training was held for the health facility staff to help them get acquainted with the modifications made and to address the data quality issues that had been identified in the evaluation.
**vi. Reporting:** ILI surveillance data in the country is reported on a weekly basis. The team agreed on a weekly-aggregated COVID-19 results in the same format and frequency as they have been reporting influenza surveillance data. Also, a real time reporting of COVID-19 should be done using the SORMAS reporting tool. Measures were outlined to ensure surveillance staff at various public health reference laboratories, veterinary laboratories and sentinel sites were trained on the use of SORMAS as a reporting tool for COVID-19.

**Fig 2:**
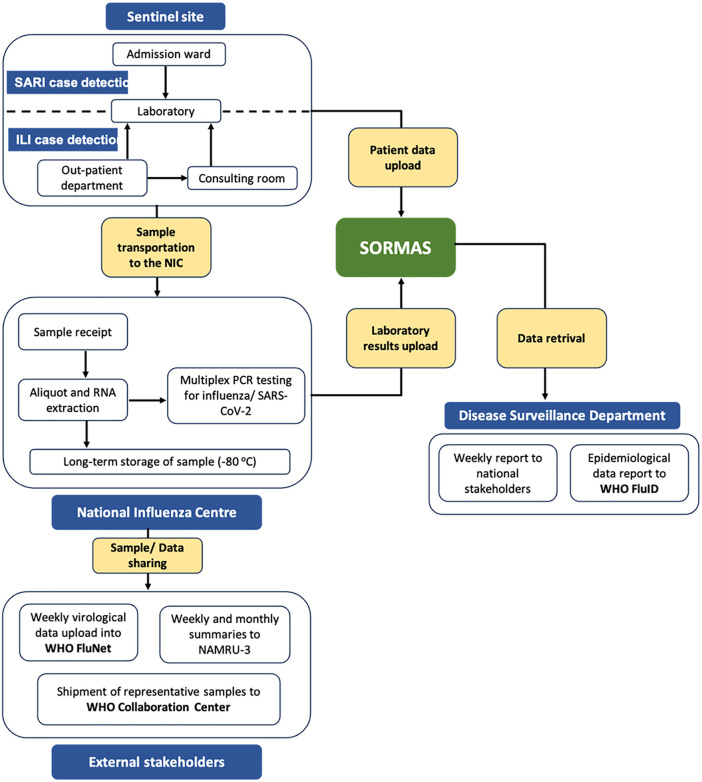
Flow chart of the integrated of SARS-CoV-2 into the ILI/SARI surveillance system. After the integration, all samples received were processed for both influenza and SARS-CoV-2.

After the integration of SARS-CoV-2 testing into the ILI/SARI surveillance system, work flow for the system was reviewed. Samples received were tested simultaneously for both influenza and SARS-CoV-2 using a US CDC primer probe set that detects both pathogens (Fig 2).

### Piloting ILI/SARS-CoV-2 testing in sites

During piloting, a total of 488 samples were received from 24 health facilities, 34 tested positive for influenza while 83 tested positive for SARS-CoV-2 (Table 2).

**Table 2:** Samples tested for influenza and SARS-Cov-2 at NIC from April – June, 2021.

Month	No. of Samples Received	No. of Samples Processed	Total Influenza Positives	SARS-CoV-2 Positives
April	211	211	17	39
May	198	198	7	37
June	79	79	10	7
**Total**	**488**	**488**	**34**	**83**

#### Roll-out ILI/SARS-CoV-2 testing to other sites.

**Fig 3:**
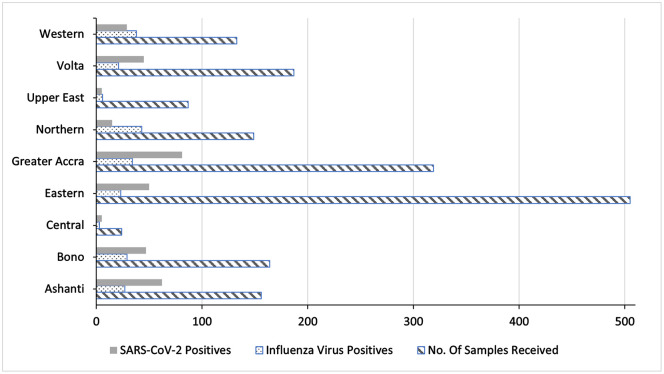
Samples tested for influenza and SARS-Cov-2 at NIC by region from July – September, 2021.

**Fig 4:**
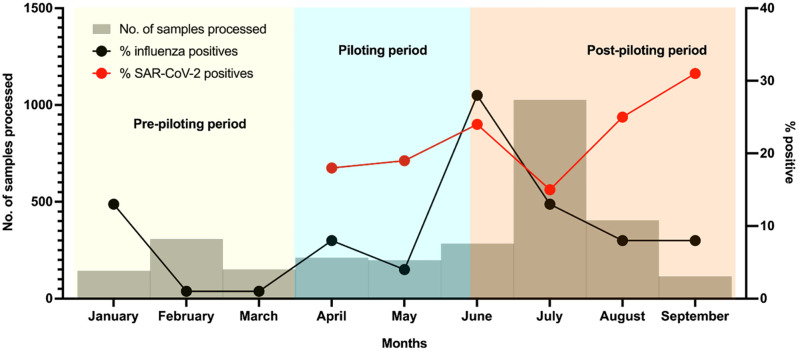
Trends of influenza virus and SARS-CoV-2 detection pre-piloting, piloting and post-piloting period.

Integration of the ILI/SARS-CoV-2 surveillance system was rolled out to all 29 sentinel sites in June 2021. Samples collected from the sentinel sites were transported and received by the lab within 48 hours and upon receipt were tested within 24 hours. From June 16 to September 10, a total of 1724 suspected influenza samples were received and tested for SARS-CoV-2 of which 339 tested positive for SARS-CoV-2 (Fig 3). Table 3 shows comparisons of samples processed during pilot and post roll out periods. We observe an increase in number of samples processed and SARS-CoV-2 detections after the full roll out. Fig 4 shows trend analyses of influenza and SARS-CoV-2 positivity across pre pilot, piloting and post full roll out phases. This trend analyses shows higher positivity rates for SARS-CoV-2 compared to influenza, an indication that case definition was sensitive to COVID-19 cases as well.

**Table 3:** The number of samples submitted and the number of positives detected for influenza virus and SARS-CoV-2 during the pilot study and post full roll out.

Syndrome	Durations	Month	No. of samples received	No. of samples processed	Influenza positive	SARS-CoV-2 positives
ILI	Pilot study	April	183	183	15	36
		May	171	171	6	35
		June	68	68	9	8
	Post-pilot study	June	132	132	42	39
		July	938	938	112	141
		August	353	353	30	84
		September	97	97	7	33
**ILI Total**			**1942**	**1942**	**221**	**376**
SARI	Pilot study	April	28	28	2	2
		May	27	27	1	2
		June	11	11	2	0
	Post-pilot study	June	49	49	18	13
		July	88	88	26	10
		August	50	50	4	17
		September	17	17	2	2
**SARI Total**			**270**	**270**	**55**	**46**
**Overall**			**2212**	**2212**	**276**	**422**

The piloting period was from 1^st^ April to 10^th^ June, 2021, and the post-piloting period is from 16^th^ June to 10^th^ September. Of the 29 sentinel sites, 16 submit ILI and SARI samples while 13 submit on ILI samples. Of the samples processed, SARS-CoV-2, positivity rate was 19.5%.

#### Sustainability steps incorporated in the integrated ILI surveillance system.

For sustainability, the health staff involved running in the integrated system at the sites are government workers carrying out the surveillance activities as part of their routine work. Similarly, the NIC operates in the NMIMR which is a government medical research center with dedicated staff receiving technical support from the Ghana Health Service Disease Surveillance Department to implement the integrated system. Therefore, in the face of declining donor funds, the system is set to run at least operate at it optimum level using the existing resources available.

## Discussion

With knowledge of the ILI/SARI surveillance system and stakeholder collaborations, Ghana integrated COVID-19 testing into the ILI/SARI surveillance. Understanding the surveillance of Influenza Like Illnesses, and improving upon it, especially in an era of emergence of novel strains including the SARS-CoV-2, has contributed to reducing the public health impact of the syndrome. Considering the core objective of integration of the Integrated Disease Surveillance & Response (IDSR) System as adopted by Ghana, the existence of a surveillance system which COVID-19 surveillance which has been fit into serves a great advantage. The recommendation by the WHO calling for countries to integrate COVID-19 testing into the existing ILI/SARI surveillance system was not just timely but an important step in getting ahead of the condition [10].

The initial evaluation of the existing surveillance system revealed that the system was partially meeting its objectives, reporting unusual events which indicate changes in circulating subtypes of influenza to WHO, informing outbreak and pandemic detection and the necessary chemoprophylaxis action. Additionally, the simplicity, flexibility, stability and adaptability of the nationwide system were advantageous in the process of integration [15–17]. The integration was fruitful because of the ability to leverage on the similarities in the existing system. For example, the same stakeholders from the lowest to the highest level were needed in COVID-19 surveillance. Also, the same nasopharyngeal and oropharyngeal specimen collected through the ILI surveillance system were used for SARS-CoV-2 testing. This made changes such as modifications to case-based forms simple. Additionally, stakeholder knowledge of the case definition and being adequately abreast with case detection of COVID-19 [18] facilitated the possibility of adapting the WHO recommendation on integration of the systems to countries [10]. This approach Ghana used is similar to the approach used by the Pan American Health Organization (PAHO) Flu System and Madagascar in their integration of SARS-CoV2 into their existing Influenza and SARI surveillance. PAHO held virtual meetings with stakeholders [19].

Stakeholder understanding of the system and their willingness to collaborate was instrumental in the integration. The COVID-19 pandemic taught clearly the lesson of collaboration in solving public health problems [20]. The ability of the various stakeholders such as researchers, health workers, other health experts and people directly involved in the running of the ILI/SARI surveillance system to agree and work together explains the integration. The process of integration saw collaboration from the national through to the grassroot levels. At the lowest levels, staff in the health facility actively participated in the evaluation, and modification of reporting forms. The laboratory staff willingness to be trained and test ILI samples for SARS-CoV-2 and fruitful dialogue among all stakeholders in the country buttress the importance of multisectoral collaboration in handling public health issues. A similar approach was used in Madagascar, where reporting tools were updated and staff at the various levels including sentinel sites were also involved in the integration process [19].

At this stage of integration where the pilot has been expanded to the various sites across the country, assessing sustainability steps to ensure the integration continues is of essence. With the current approach, sustainability of the integration has been ingrained into the entire process. At the facility level, health workers take the same samples and fill the modified forms which have currently replaced the ILI base-based forms that were used. This process forms part of their routine activities. As a way of dealing with the data quality challenges identified in the surveillance evaluation, health workers were retrained on how to fill the forms and the need to provide information for all the sections of the form.

Sample transport remains the same using the system already in place. Finally, the system receives regular samples which are also tested for SARS-CoV-2 as the part of the routine testing for ILI/SARI samples. The process of integrating COVID-19 surveillance into the existing ILI/SARI surveillance system in Ghana demonstrates the strength in multisectoral collaboration, and leveraging on existing systems in solving public health situations which continue to happen unexpectedly.

To ensure the integrated system is sustainable, the integration process leveraged on already available human resources capacity and existing structures. this approach strengthens the system and makes it stable even in the face of dwindling financial resources.

### Public Health implications on integration of COVID-19 testing

Fruitful implementation of COVID-19 testing into the ILI/SARI surveillance system has several public health implications going forward. Due to the flexible nature of the surveillance system and the fact that it is able to serve its purpose of monitoring these two pathogens among the general population, other pathogens such as human respiratory syncytial virus and other respiratory viruses of pandemic potential (coronaviruses) could be integrated. This will enable monitoring of multiple pathogens of pandemic potential. Again, such an integrated system will serve as an early detection warning system for future epidemics. For low- and middle-income countries such as Ghana, with limited funding, such an integrated system will be easy to operate and provide tremendous benefits to the country. These lessons from ILI surveillance could serve as a step for other surveillance systems to identify potential points and ways of leveraging on the existing systems to meet the changing epidemiological needs of the population.

Some operational challenges encountered during implementation were as follow; Implementation of this integrated approach came with certain challenges. One identified limitation was the transfer of healthcare staff involved in the integration process to other health facilities, slowing reporting as new staff familiarise themselves with the new assigned tools. Also, there were some delays between data collection and entry into the reporting system (SORMAS), affecting real-time response.

Some potential biases and limitations to surveillance reporting were poor data quality due to poor record keeping and unavailability of staff at the facility level to take part in surveillance activities. This puts a strain on the few available staff willing to take on ILI surveillance. Since they are burdened with its responsibilities, they are unable to keep good records at the lower level. Despite these challenges, this paper shows the ability of countries to leverage on existing systems to meet the changing needs of the health system.

## Conclusion

The flexibility of the ILI/SARI surveillance system in Ghana aided the integration of the COVID-19 surveillance into the existing system. The similar case definition, samples used for testing, testing mechanisms, and the epidemiological data collected facilitated the integration of COVID-19 testing into the ILI/SARI surveillance systems. The integration of SARS-CoV-2 in the influenza surveillance system is of great benefit to public health. This will help establish seasonality of SARS-CoV-2, set baseline levels and thresholds that will signal the beginning and end of epidemics, and monitor variant viruses’ transmission and impact on health systems. Also, this will help identify unexpected pattern changes relative to known experience and provide surge capacity for testing and outbreak investigation. Furthermore, data gathered will inform relevant and optimal measures for prevention and control, including antiviral treatment and vaccination. To continually sustain the gains made, prudent supervisory measures should be instituted by the Ghana Health Service and the National Influenza Center to ensure sentinel sites submit samples on weekly basis for testing.

The model in addition to its valuable lessons offered to strengthen routing surveillance can also serve as a guide for improving pandemic preparedness in similar settings.

## Recommendation

Data completeness was low; to improve on this attribute, there needs to be a periodic data audit and feedback to the sentinel sites. Also, for SORMAS, if possible, all fields must be made mandatory for demographic, clinical and laboratory data entry. We recommend the establishment of ILI surveillance teams at each of these sentinel sites instead of individuals. This will ensure improved surveillance activities at the sites even if the same designated focal persons were no longer available. Additionally, regular training for persons involved in ILI surveillance would keep stakeholders updated on the system’s activities.
